# When phylogeny and ecology meet: Modeling the occurrence of Trichoptera with environmental and phylogenetic data

**DOI:** 10.1002/ece3.4031

**Published:** 2018-05-08

**Authors:** Bruno Spacek Godoy, Lucas Marques Camargos, Sara Lodi

**Affiliations:** ^1^ Núcleo de Ciências Agrárias e Desenvolvimento Rural Univ Federal do Pará Belém Brazil; ^2^ Departament of Entomology Univ of Minnesota Saint Paul MN USA; ^3^ Programa de Pós‐Graduação em Ecologia e Evolução Univ Federal de Goiás Goiânia Brazil

**Keywords:** aquatic insects, Bayesian updating model, evolutionary ecology, predictive modeling

## Abstract

Ecological studies are increasingly considering phylogenetic relationships among species. The phylogeny is used as a proxy or filter to improve statistical tests and retain evolutionary elements, such as niche conservation. We used the phylogenetic topology to improve the model for occurrence of Trichoptera genera in Cerrado (Brazilian Savanna) streams. We tested whether parameters generated by logistic models of occurrence, using phylogenetic signals, are better than models generated without phylogenetic information. We used a model with Bayesian updating to examine the influence of stream water pH and phylogenetic relationship among genera on the occurrence of Trichoptera genera. Then, we compared this model with the logistic model for each Trichoptera genus. The probability of occurrence of most genera increased with water pH, and the phylogeny‐based explicit logistic model improved the parameters estimated for observed genera. The inferred relationship between genera occurrence and stream pH improved, indicating that phylogeny adds relevant information when estimating ecological responses of organisms. Water with elevated acidity (low pH values) may be restrictive for the occurrence of Trichoptera larvae, especially if the regional streams exhibit neutral to alkaline water, as is observed in the Cerrado region. Using phylogeny‐based modeling to predict species occurrence is a prominent opportunity to extend our current statistical framework based on environmental conditions, as it enables a more precise estimation of ecological parameters.

## 1 INTRODUCTION

The ecological niche is the key concept for understanding the occurrence and distribution of species in natural environments (Lepš, de Bello, Lavorel, & Berman, 2006). Both ecological and evolutionary sciences use this concept to elaborate theories and hypotheses to explain the distribution of biological diversity. The importance of the niche theory in structuring communities has been at the core of ecological theory since the establishment of Ecology as a science, from the formal proposal for communities (Hutchinson, 1957) to the dichotomy of niche‐based versus neutral models (Mikkelson, 2005). The niche concept has two advantages in ecological and evolutionary studies: first, its predictions may be readily used in modeling; second, it has been widely used in ecological studies (Colwell & Rangel, 2009). The niche summarizes the environmental enclosures (both the biotic and abiotic factors) within which a species can survive and maintain a stable population. This structure enables the construction of predictive species distribution models and supports comparative and synthesis studies (Araújo & Guisan, 2006; Domisch, Jähnig, Simaika, Kuemmerlen, & Stoll, 2015).

In an evolutionary context, the conservation of ancestral traits in modern species is relevant to the debate in community ecology on the factors determining species composition, given that the species’ niche may influence the biological community structure (Westoby, 2006). In the field of Evolutionary Ecology, niche conservatism explains how related species tend to occupy similar habitats (Procheş, Wilson, Richardson, & Rejmánek, 2007; Wiens et al., 2010). However, species rarely have identical niches, which leads to an imperfect niche conservatism (Losos, 2008). Additionally, the true relevance of niche conservatism, as estimated from phylogenetic studies, is not clear from community ecology studies (Cavender‐Bares et al., 2009; Kraft et al., 2015). The main relevance of considering niche conservatism in statistical analysis, in which species and other taxonomic units are otherwise treated as independent observations (Felsenstein, 1985), is to account for their phylogenetic relationship or phylogenetic autocorrelation (the main source of dependence among species; Martins & Garland, 1991; Martins & Housworth, 2002; Garland, Bennett, & Rezende, 2005).

Phylogenetic autocorrelation is perceived as a source of error in statistical inference in ecological studies. Therefore, when analyzing related groups with similar characteristics, the effect of phylogenetic relationships is usually removed (or controlled) to disentangle ecological processes resulting from common ancestry in explaining the trait in question (Diniz, 2001; Westoby, Leishman, & Lord, 1995). In the frequentist statistical framework, phylogenetic signals generate bias in ecological analysis, increasing type I error, because the differences in ecological traits among species pairs cannot be used as primary information given the phylogenetic dependence among species (Martins & Housworth, 2002). More current analytical methods are working toward using the information available in the phylogeny to measure evolutionary diversity (Tucker et al., 2017) and to model ecosystem functionality (Srivastava et al., 2012).

However, if ecological theory treats phylogenetic signals as only a source of inferential confusion, we lose relevant information on the relationship between species and the environment (Blomberg, Garland, & Ives, 2003). Phenotype–trait similarities tend to increase in more phylogenetically related species, in both behavior and morphology (Guénard, Legendre, & Peres‐Neto, 2013). This phylogeny–trait correlation could be contemplated as a signal, allowing traits of easily studied species to be used in estimating traits of phylogenetically related species that are more difficult to observe and sample (Guénard, von der Ohe, de Zwart, Legendre, & Lek, 2011; Losos, 2008). Therefore, phylogenetic signal may be correlated with niche conservatism, allowing it to be used as surrogate information on rare or uncommon species. Another advantage of using niche conservatism in phylogeny is the possibility of inferring trait change in the evolutionary history of a clade and elaborating hypothesis about the evolutionary history of the studied group.

In this study, we elaborated and tested a statistical framework based on Bayesian inference, capable of using species trait information and signal from a phylogenetic tree of Trichoptera. We proposed a multilevel model to estimate environmental and trait dependence parameters, using phylogenetic signals to improve the prediction of Trichoptera genera occurrence related to chemical characteristics of Cerrado (Brazilian savanna) streams. We chose to model the order Trichoptera because the phylogenetic relationships of its genera are relatively well studied up to high taxonomic groups (e.g., family level) compared to other neotropical aquatic insects (Kjer et al., 2016). In addition, many studies indicate that occurrence of Trichoptera species depends on environmental conditions (Couceiro, Hamada, Ferreira, Forsberg, & Silva, 2007; Couceiro, Hamada, Forsberg, & Padovesi‐Fonseca, 2010; Cuffney et al., 2011). Another advantage of using an animal clade is that phylogenetic signals frequently show influence in contemporary ecological and behavioral traits (Harmon et al., 2010; Dias et al., 2009), providing good models to test phylogenetic relationship related to environment dependence.

The objective of this study was to test the effects of the environment on the occurrence of Trichoptera genera in Cerrado streams, using phylogenetic signals to improve predictions. We used the variable pH to indicate the environmental modifications. We analyzed genera occurrence in two scenarios: with or without the phylogenetic signals determining the parameters of relationship with the environment and species occurrence. Accordingly, we determined the extent to which using evolutionary information provided by phylogenetic tree improves inferences derived from ecological studies, allowing better estimation of statistical parameters related to environment occurrence–dependence of Trichoptera genera.

## 2 METHODS

### 2.1 Study area and sampling methods

**Figure 1:**
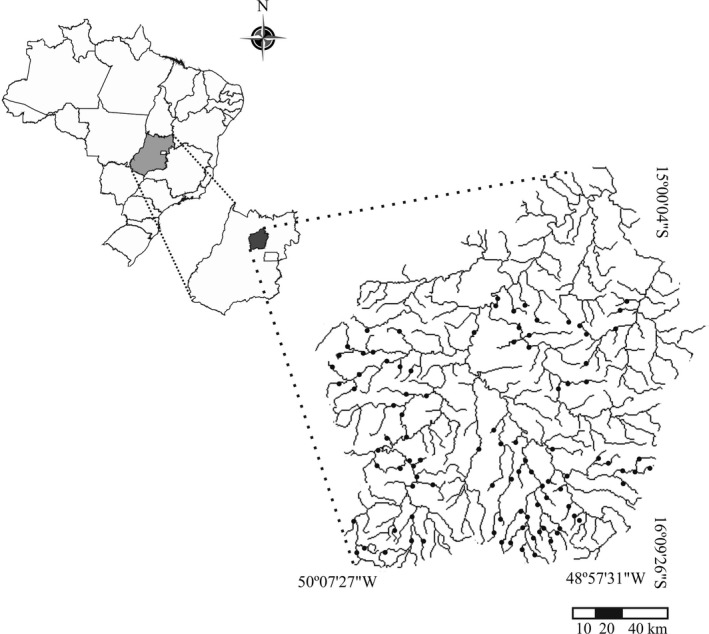
Distribution of the sites where the Trichoptera genera were sampled, within the Rio das Almas basin, Brazil

We sampled 101 streams or rivers in the Rio das Almas basin (central region of the state of Goiás, Brazil) during the months June and August of the year 2009 (Figure 1). We sought to sample the largest possible range of river size variation (average width = 3.18 ± 3.14, width range 0.44–16.75 m) to reflect the natural heterogeneity. This river basin has both well‐preserved and degraded areas (Godoy, Simião‐Ferreira, Lodi, & Oliveira, 2016). Intensive agriculture, livestock activities, highly deforested areas, and siltation prevail as the main types of habitat degradation (Godoy et al., 2016). According to the Köppen classification, the climate in the study region is tropical Aw, with a dry period of 5 months (May to September), and mean annual temperatures ranging from 24 to 28°C (maximum and minimum temperatures ranging from 29 to 33°C and 18 to 22°C) (Peel, Finlayson, & Mcmahon, 2007). Annual precipitation in this region ranges from 1650 to 1850 mm (INMET 2015). Samples were collected only during the dry period, when the sampling efficiency for Cerrado streams is highest, due to a lower removal of individuals by the rainwater (Bispo, Oliveira, Crisci‐Bispo, & Sousa, 2004).

We sampled the Trichoptera individuals in four different microhabitats, namely marginal vegetation, stones, bottom litter and sand, using a 0.025‐mm hand sieve, for 15 min in each microhabitat. The use of hand sieve reduces the amount of debris in the samples, so less time is spent separating insects from debris. In addition, this mesh size is useful for retaining late instar larvae, making the process of specimen identification easier and faster. We selected microhabitats from a stream segment of 100 m and randomly sampled individuals from each microhabitat. We chose this sampling method because it provides an estimate of community composition parameters similar to more exhaustive sampling procedures (Chiasson, 2009). The specimens were identified following Pes, Hamada, and Nessimian (2005). We calculated the arithmetic mean of five pH measures portray this chemical characteristic of the water, before conducting the biological sampling. We used only pH because previous empirical evidence has shown a close correlation between ionic content of water and stream biota (Allan & Castillo, 2007), and due to its great variability in the study area.

### 2.2 Obtaining phylogenies

We obtained phylogenetic trees from the literature and combined all the information in a single supertree. We used two caddisfly phylogenies, where each used both morphological and genetic traits. The first phylogeny comprised sequenced fragments of large‐subunit and small‐subunit nuclear rRNAs (1078 nt; D1, D3, V4‐5), the nuclear gene elongation factor 1α (EF‐1α; 1098 nt), a fragment of mitochondrial cytochrome oxidase I (COI; 411 nt), and seventy adult and larval morphological characters (Kjer, Blahnik, & Holzenthal, 2001). The second phylogeny used the same molecular and morphological characters, but different weighting methods for parsimony analyses, and included families in the tree (Holzenthal, Blahnik, Kjer, & Prather, 2006; Kjer, Blahnik, & Holzenthal, 2002).

We used the Robinson–Foulds distance to create topological structure for the supertree (Davis & Hill, 2010). In our study, we disregarded branch lengths for the tree topology and retained only genus‐level information. Our occurrence model uses phylogeny only as a proxy to determine the inclusion of taxon parameters into estimations. We avoided including the strength of the phylogenetic autocorrelation between genera and higher taxa in the model, so we did not use branch lengths of the tree, once these lengths represent the hypothetical parental distance among taxa.

### 2.3 Elaborating a phylogeny‐based explicit model for occurrence

We used Bayesian updating to investigate the influence of water pH on the occurrence of Trichoptera genera. The model was based on how easily Bayes’ theorem combines probabilistic information from different sources, if that information has a hierarchical structure and if it provides a logical and consistent framework to explore evolutionary traits. Using prior and posterior distributions, linked with exhaustive iterative methods to estimate the parameters, facilitates the analytical structure, as likelihood optimization is applied to each studied parameter and not to all elements in the analysis simultaneously, as in other inferences (Stamps & Frankenhuis, 2016).

The model assumes that before being exposed to an environmental condition, a genus has a predetermined response to the environmental variable, based on information from its ancestor taxon. These predetermined responses are then revised according to the response of the genus, after it is exposed to the environmental condition. The model easily accommodates situations with several levels of cladogenesis in a phylogenetic tree, where only one hierarchical scheme is required for inference. The result of the model is the posterior distribution for each genus and each cladogenesis event in the tree. Therefore, the response of each genus is affected by the environmental conditions and a possible response from an ancestor. A relevant element in this model is the possibility of improving parameter estimation, even when the number of samples is small for the taxon studied. This is possible as the parameter of the proximal ancestor should be estimated by the taxon with the most occurrences, sharing the same phylogenetic branch, and originated from the same cladogenesis event.

The model was based on a logistic regression, but parameter estimation considers the environmental information of the sites where the genera occur and the phylogenetic signal of the nearest genus in the phylogeny topology. We chose to use the logistic regression because this analysis was the most used in population ecology of aquatic insects, especially in studies regarding occurrence (Iversen et al., 2016). The logistic regression considers the environmental limits for the occurrence of the organism and is therefore a simple model to predict its distribution. This model has been applied in aquatic ecology, once it provides a result similar to that of other mathematical associations between organismal occurrence and chemical water variables (Hole, 1998), such as unimodal or multimodal distributions. The formal model we present has the following configuration:

$$\begin{array}{cc} & O_{i,j}∼\text{Bernouli}(P_{i,j})\\ & \text{Logit}\left(P_{i,j}\right)=\alpha_{j}+\beta_{j}\cdot x_{i}\\ & \alpha_{j}∼\text{Normal}\;(\text{mean}=0,SD=1.0\times 10^{-6}\\ & \beta_{j}∼\text{Normal}\;\left(\text{mean}=\beta_{j-1},SD=\tau_{j}\right)\\ & \tau_{j}∼\text{Gamma}\;\left(0.001,\;0.001\right)\\ & \sigma_{j}=\frac{1}{\sqrt{\tau_{j}}}\\ & \cdots \\ & \beta_{1+1}∼\text{Normal}(\text{mean}=\beta_{1},SD=\tau_{1+1})\\ & \beta_{1}∼\text{Normal}\;\left(\text{mean}=0,SD=\tau_{1}\right)\\ & \tau_{1}∼\text{Gamma}\;\left(0.001,0.001\right)\\ & \sigma_{1}=\frac{1}{\sqrt{\tau_{1}}} \end{array}$$

**Figure 2:**
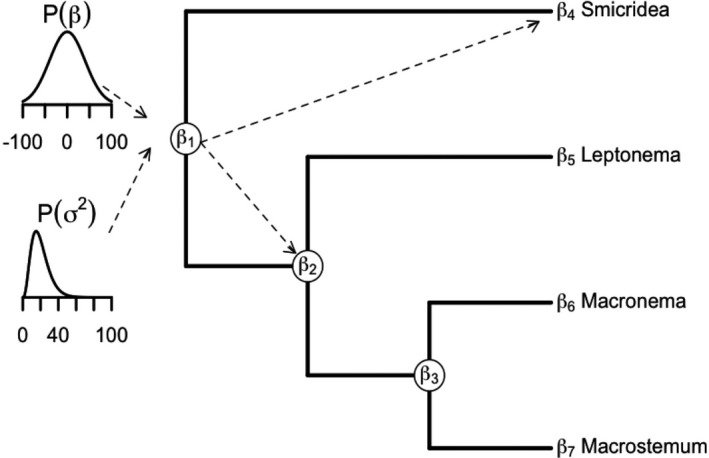
Illustrated example of the phylogeny‐based explicit logistic model for estimating the parameters. We started in the basal node of phylogeny (β_1_), and the other parameters are estimations made from this primary distribution

In this model, the occurrence of the genus *j* in a stream *i* (*O*_*i,j*_) was conditioned to the environment, in our case, pH (*x*_*i*_). This logistic model structure is the same in the usual analysis, but the parameter of dependence with environment (β_*j*_) was taken to another parameter distribution (β_*j*−1_), related to a possible ancestor of the group (Figure 2). This logic was applied to each cladogenesis event, up to the first node of the phylogenetic tree, where the prime parameter of the environmental relationship is estimated (β_1_). We used an a priori distribution for the prime parameter with zero mean and high variance (1.0 × 10^−6^), and all precision estimators in the model (τ_._) had an uninformative gamma distribution as an a priori distribution. The last equation is required to estimate variance, as it is the inverse of precision. We used the Markov chain Monte Carlo (MCMC) with five independent chains to estimate the a posteriori distribution of the parameters. We sampled in intervals of five iterations to avoid dependent estimates and used 1,000 iterations in each chain after discarding 1,000 “burn‐in” iterations (calibration period).

### 2.4 Testing the improvement of the phylogeny used in the occurrence model

We modeled the occurrence of the Trichoptera genera twice, with and without the phylogeny as a proxy for niche conservatism. In both analyses, we used the Bayes decision regarding hypothesis acceptance and compared the credibility of parameters for both models (with or without the phylogeny in the logistic regression model). Bayes decision is obtained by estimating the posterior probability of rejecting a lack of effect of a parameter and that probability is calculated by reducing a predetermined inferential prejudice when we choose a hypothesis over a simpler one (Pawlak, 1999).

In our study, we may determine different decision ranges for whether we should accept the estimated parameter, given that we calculated a decision probability. We accepted the parameter with a very strong inference when the probability of rejecting the lack of effect was lower than 0.05 and accepted the parameter with a strong inference for a probability value ranging from 0.05 to 0.15. The inference becomes only acceptable for probability values ranging from 0.15 to 0.30, when caution in result interpretation becomes necessary. If the probability exceeds 0.30, there is little evidence to accept the given studied parameter. The method used to develop different decision ranges in hypothesis testing agrees with the Neyman–Pearson lemma and enables the retention of relevant information on the data (Paulino, Turkman, & Murteira, 2003). All analyses were carried out using the BRugs package in the statistical software R (R Development Core Team 2014).

### 2.5 Model validation

We used a cross‐validation procedure to evaluate the performance of the estimator and the accuracy of model prediction in practice. In each round of cross‐validation, we partitioned the data into two complementary subsets—the training set and the validation set. The validation set corresponded to 5% of genera observed in the study, contrasting with the conventional use of 30% of the total data. We chose to use only 5% of the data for two reasons: first, due to the limited number of Trichoptera genera sampled in the streams; second the removal of a relatively high number of genera from a phylogeny may change tree topology, modifying several elements such as number of nodes and phylogenetic relationships among genera. Therefore, removing a restricted number of genera from the dataset to use in validation preserved the topology of the phylogenetic tree.

We iterated the procedure 1,000 times and always randomized the subset of genera allocated to validation. We chose to use a higher number of iterations than the possible groups of validation sets, once the parameters estimated in the model are a result of MCMC. Thus, the MCMC is generated in each iteration, a vector of parameters not necessarily equal to that of other iterations with the same data. This property of the analyses allows the inclusion of random processes in the model even when a limited validation set is sampled, such that estimations may be validated and tested.

In each iteration, we removed the validation set and estimated the parameters of the model with the training set. After estimating the parameter, we retained the estimated β values for the nodes closer to the genera in the validation set and used the β parameter to calculate the expected occurrence for the genera chosen for validation. We used the expected occurrence to calculate the area under the curve (AUC) based on the ROC curve of model accuracy and prevalence (Lobo et al., 2008). The AUC statistics allows us to determine whether the model was able to estimate genera occurrence using only the phylogenetic information, which in our study is the estimated β for the nearest node. The AUC ranged from zero to one, and we considered values larger than 0.5 to represent a good fit. After completing all 1,000 iterations, we used the AUC values calculated in each iteration, estimated the 95% confidence interval for this statistic, and tested the ability of the model to predict occurrence of genera.

## 3 RESULTS

We sampled on average 5.61 (±2.58) genera of Trichoptera per stream and 2,027 individuals (30.71 ± 26.26 per stream), referring to 24 genera of Trichoptera. The streams surveyed exhibit neutral to alkaline water, a normal pattern for the Cerrado region (pH 7.29 ± 0.92, ranging from 3.72 to 8.80). The most common and abundant genera were *Smicridea* and *Leptonema*, while *Anchitrichia* and *Atopsyche* were the most rare (Table 1). *Atopsyche* and *Cyrnellus* exhibited the lowest abundance.

**Table 1:** Occurrence and abundance of each Trichoptera genera sampled in the Rio das Almas river basin

Genus	Occurrence	Abundance
*Alisotrichia*	3	3
*Anchitrichia*	1	65
*Atopsyche*	1	2
*Austrotinotes*	3	5
*Cernotina*	8	12
*Chimarra*	31	288
*Cyrnellus*	2	2
*Helicopsyche*	29	219
*Itauara*	4	18
*Leptonema*	46	306
*Macronema*	34	132
*Macrostemum*	19	100
*Marilia*	27	144
*Nectopsyche*	14	60
*Neotrichia*	3	3
*Ochrotrichia*	15	31
*Oecetis*	16	42
*Oxyethira*	7	72
*Phylloicus*	27	121
*Polyplectropus*	5	8
*Protoptila*	5	7
*Smicridea*	47	338
*Triplectides*	18	42
*Zumatrichia*	5	7

**Figure 3:**
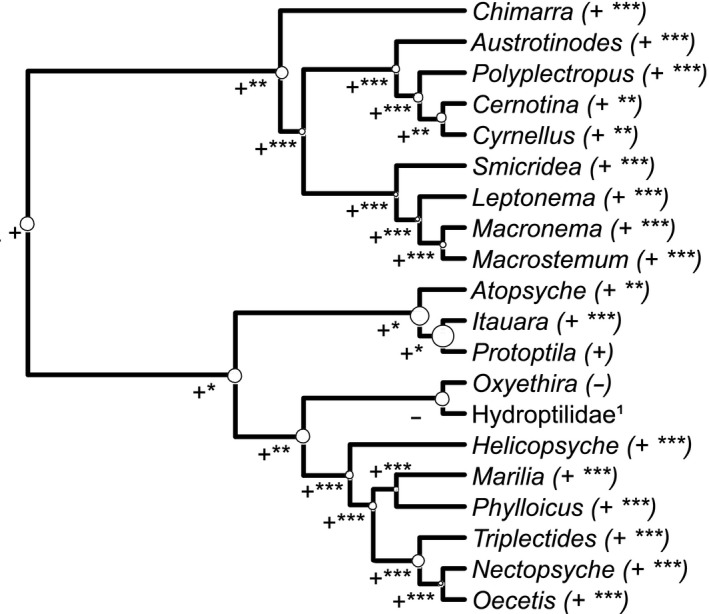
Phylogeny of Trichoptera genera observed at Cerrado streams in Brazil. The positive or negative signals indicate the sign of the beta parameter estimated. The size of circles represents the variance for the parameter estimated, and stars indicate the inference for the estimates, * acceptable parameter, ** strong inference to accept the parameter, and *** very strong inference. ¹The Hydroptilidae genera were grouped because the estimated parameter did not have enough inference

All taxa, except Hydroptilidae, had their occurrence influenced by water pH, and probability of occurrence for most genera increased in alkaline water (Figure 3, positive values for the parameter beta). The genus *Protoptila* and the family Hydroptilidae were exceptions, as the parameter of dependence with pH was positive and the model indicated a nonrelevant relationship. A relevant result we present is the parameter estimated for the nodes of the phylogeny, which indicates a possible ancestral relationship with environment. Our result shows that Neotropical Trichoptera prefer more alkaline water, for both modern and ancestral genera (Figure 3). The direct ancestor of Hydroptilidae and *Protoptila* had a high variance in the estimated relationship with the environment. However, the ancestor of *Atopsyche*,* Itauara,* and *Protoptila* had the highest variance for this parameter in the phylogeny.

**Figure 4:**
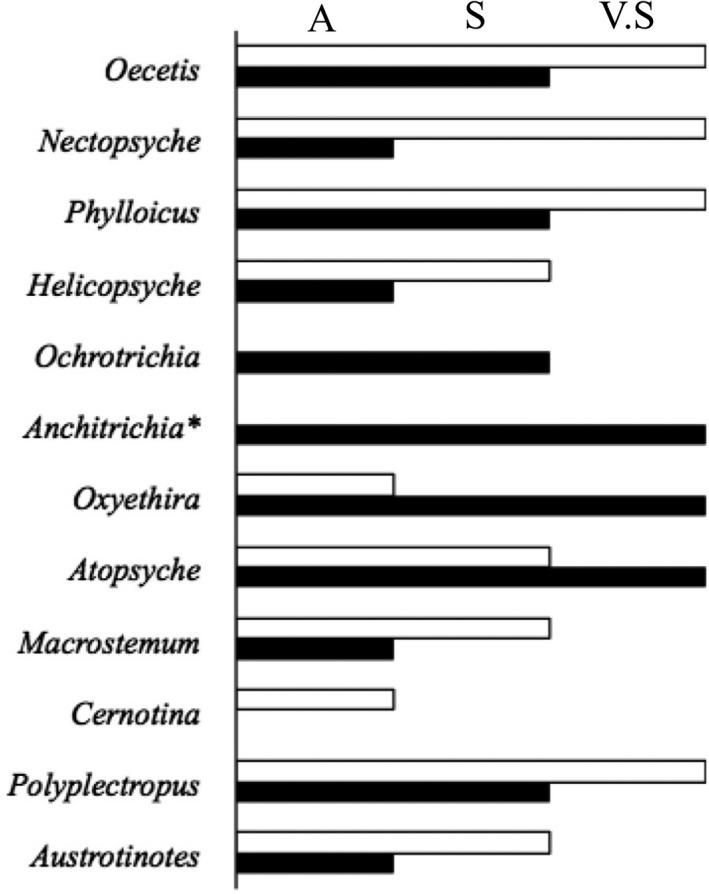
Bayes decision regarding the inference of the parameter beta using the phylogeny‐based explicit logistic model (white bar) in contrast to a simple logistic model for the occurrence of Trichoptera genera (black bar). A, acceptable; S, strong; V.S, very strong. *Anchitrichia changed the parameter sign to negative from positive

The use of the phylogeny‐based explicit logistic model improved parameter estimations for the genera (Figure 4). The inference of accepting the relationship between genera occurrence and stream water pH was improved, indicating that the phylogeny added relevant information to the evaluation of ecological response of organisms. For the genera *Anchitrichia*,* Ochrotrichia* (Hydroptilidae), and *Atopsyche*, using phylogenetic signal weakened parameter estimation. However, these genera had low occurrences in the sampled streams (one occurrence for *Anchitrichia* and one for *Atopsyche*). The use of phylogenetic information may be in fact correcting spurious relationships and deflecting from incorrect conclusions.

### 3.1 Model validation results

**Figure 5:**
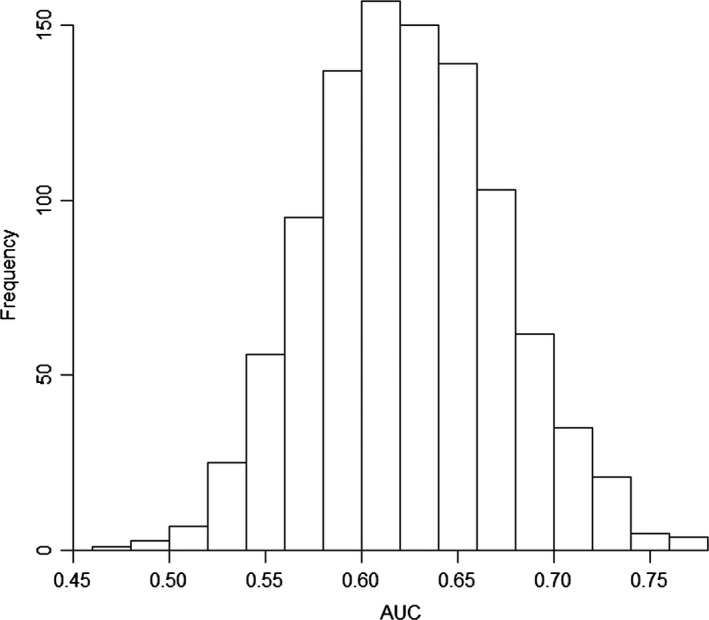
AUC calculated for the expected occurrence of genera against the observed occurrence of the genera

The model was consistent in estimating the occurrence of the genera in the validation set. The AUC statistic for the 1,000 iterations was higher than the 0.5 threshold established to validate the model (AUC mean = 0.62, with the 95% CI ranging from 0.53 to 0.73, Figure 5). Lower AUC values were recorded for the genera of Hydroptilidae family (AUC = 0.47). The genera *Chimarra*,* Smicridea,* and *Leptonema* had the highest AUC values (about 0.77). These results may be related to the high occurrence of these genera, as *Chimarra*,* Smicridea,* and *Leptonema* occurred in many of the sampled streams, and Hydroptilidae genera were rarest in the study area.

## 4 DISCUSSION

### 4.1 Model performance

The great novelty of our study is the Bayesian model built for occurrence prediction, evaluating phylogenetic and survey evidences. Our model uses information on the phylogenetic relationships among genera directly, avoiding the use of indirect inferences, such as multivariate ordinations (Guénard et al., 2011). The direct use of phylogenetic topology does not depend on the number of species used in the construction of the phylogenetic tree to provide auxiliary information on phylogenetic conservatism. Therefore, the relationship between dependence of the trait of an organism and the environment can be analyzed directly in the phylogenetic topology. The great advantage is the possibility of an interchange of information among taxa, reaching more accurate parameter estimation. In such approach, the phylogenetic information is used not only to correct phylogenetic autocorrelation, but also as an auxiliary inference for ecological studies. However, caution is needed when using the phylogeny‐based explicit model, as the intensity of phylogenetic autocorrelation in the trait studied creates phantom dependence between a genus and the environment, mainly in genera with very reduced occurrence events (Guénard et al., 2011).

Our method does not depend on the number of species in the analyses, but the model is strongly related to the topology of the phylogeny. An outcome of this dependence is the increased number of parameters in the model for each node added to the phylogenetic tree. The increased number of parameters contradicts the principle of parsimony, improving the model fit because of the many parameters estimated. Note that a lack of parsimony may surface idiosyncrasies of the data rather than make the underlying pattern clearer. However, we are able to account for the parameters estimates in our model, where estimating many parameters for environmental dependence within the phylogeny nodes at the tree allowed us to understand the historical ecological processes during the clade formation. The parameter estimate for the tree nodes indicates a possible relationship between the ancestor and the environment. Node parameters with high variance may indicate divergence in the use of the environment among phylogenetic branches and possibly incomplete niche conservatism in the trait studied, with major niche changes occurring in some lineages. This information along with phylogeographic studies can provide a major overview on the evolution of ecological responses in the clade studied, making the increased parameter estimation in the model acceptable. Using the phylogeny to model the occurrence of Trichoptera genera is a valuable opportunity to extend our current statistical framework to estimate species occurrence based on environmental conditions. This novelty of using phylogenetic information to improve the ecological parameter has promoted a new look toward phylogenetic autocorrelation, usually used in ecological studies only to remove evolutionary dependence in related taxa (Ackerly, 2009; Ackerly & Nyffeler, 2004; Diniz, 2001).

Bayesian updating may provide great improvements to analytical tools in community and population ecology, as shown in our results. We used the priori update related to phylogenetic relationship between taxa, assuming the directional drive in cladogenesis events and the possible effects in the ecological traits studied (in our study, the occurrence of Trichoptera genera). The main logical advantage of this approach is to deflect from using nil null hypotheses (Johnson, 1999; McCarthy, 2007). Brownian motion is normally used to represent the random process in the evolutionary taxon history (Blomberg et al., 2003; Diniz, 2001; Maddison & Slatkin, 1991), and any deviation from this pattern of motion is evidence of directional environmental selection. However, the use of nil nulls may be incorrect or not provide useful scientific information in ecological/evolutionary studies (Stephens, Buskirk, Hayward, & Martínez Del Rio, 2005). The direct use of phylogenetic information in the Bayesian updates provides directional null hypotheses in each phylogeny node, constructing a reasonable prediction of evolutionary history (in this case, evolutionary history of Trichoptera).

The possibility of estimating a coefficient of relationship between the environment and occurrence of an ancestor clade in the phylogeny is not a trivial result. This information, despite being primarily an approximation, enables elaboration of hypotheses concerning Trichoptera groups and the evolutionary process of cladogenesis, and guides new experiments and observations to understand the diversification of this group. In Trichoptera phylogeny, almost every parameter estimated regarding the influence of pH on taxon occurrence showed the same pattern. The most variable clade was the group formed by Glossosomatinae and Protoptilinae (Holzenthal et al., 2006; Kjer et al., 2016), wherein *Protoptila* was the only genus that did not depend on pH for occurrence. That variability was transferred to a superior node in the phylogeny, indicating a divergence in habitat use for these genera, and a possible exploitation of unoccupied environments relative to Trichoptera species. Previous studies showed a high abundance of *Protoptila* in both rainy and dry seasons (Vázquez‐Ramos, Guevara‐Cardona, & Reinoso‐Flórez, 2014), which could also reflect a wide niche breadth of the genus as a whole. It does not mean that individual species of this genus occur in many different habitats and, without higher taxonomic resolution, it is not possible to determine which of the 13 described (and probably many other undescribed) *Protoptila* species from Brazil are being sampled in different stream habitats (Santos et al., 2016). The hypothesis that *Protoptila*, or its individual species, have a wide niche breadth needs to be tested with more specific experiments on their life history.

### 4.2 Trichoptera–environment relationship

The results of our study show the importance of environmental characteristics (specifically water pH) for the occurrence of Trichoptera genera in Cerrado streams. We highlight that the study only considered part of the niche structure of the genera (i.e., water pH). However, despite this variable being relevant for the community structure in Cerrado streams (Godoy et al., 2017), we need caution in exploring the results. Most genera occurred in streams with alkaline water. This result agrees with other studies concerning aquatic insects in the Brazilian Cerrado (Godoy et al., 2016), but their results showed an increase in the number of genera for the entire community, in contrast to our results. Water with elevated acidity (low pH) may be restrictive for the local biota, especially if the regional streams exhibit neutral to alkaline water, as in the Cerrado region (Markewitz et al., 2006). Investing in the colonization of uncommon habitats may be a good local strategy for a population, but it may limit a possible dispersal of offspring on a regional scale. Such dispersal limitations have a negative trade‐off cost for the species and lead to a reduced number of individuals and genera inhabiting acid streams.

Low pH values require physiological adaptations for the organisms to avoid deleterious effects of the acid water (Harrison, 2001). The shelter‐making behavior of many genera in this Order is another relevant specific interference of pH on Trichoptera populations. Acid water may impair the physical integrity of compounds used by the larvae to build the shelters, portable cases and cocoons, as the reduced pH has a high potential of oxidizing and corroding organic structures (Ashton, Taggart, & Stewart, 2012; Hatano & Nagashima, 2015; Stewart, Ransom, & Hlady, 2011). pH reduction was the main causal factor in an increased mortality of zebra mussel (*Dreissena polymorpha*), as available calcium is reduced in acidic water (Hincks et al., 1997).

We must, however, consider the identification of all individuals at the same taxonomic resolution (i.e., genus), as identification to species level was unfeasible given the state of knowledge of Trichoptera and other aquatic insects of tropical regions (especially larvae; Santos et al., 2016; Holzenthal & Calor, 2017). This taxonomic level could lead to a misinterpretation of results, once many genera may have several species, which could respond differently to environmental conditions (Bailey, Norris, & Reynoldson, 2001). In addition, the hand‐sieve sampling method may have underestimated the abundance of groups that are smaller and not easily removed from the substrates by hand, also explaining the low overall abundance per sampling (Holzenthal, Thomson, & Ríos‐Tourma, 2015).

Another problem is the imprecise phylogenetic relationship between taxa, such as the group formed by *Atopsyche, Itauara,* and *Protoptila*. If the cladogenesis of the group is not resolved, the location of the hyperparameter of the model may aggregate taxa with independently distinct environmental responses, inflating estimation of the parameter variance. However, we used the genus‐level identification to assess the evolutionary history of the group and assumed all monophyletic. We expected that the response of occurrence to environmental conditions would be phylogenetically autocorrelated, so the parameter estimation reflects the general pattern for the studied group. In short, we expected that identification to the genus level would have some, however, little influence on the results regarding the occurrence dynamics and estimated historical evolution of the relationship between occurrence and environment.

### 4.3 Future advances for explicit phylogenetic models

The direct use of phylogenetic information on occurrence models could be a promising tool for researchers to estimate more precise ecological parameters. It improved the estimated influence of pH on the occurrence of one‐third of the Trichoptera genera. Furthermore, the phylogeny‐based explicit model corrected estimates for genera with low occurrence, avoiding spurious results created by inappropriate sampling. Such correction is based on the information of phylogenetically close genera, providing more concise inferences.

The flexibility of the model allows the creation of a productive and effective framework for ecological data analyses. Its structure supports the use of other types of relationships, such as linear dependence (linear regression) and multinomial responses. We can model other responses, such as frequency of occurrence or abundance, colonization success, and other ecological variables, changing the likelihood of the model (the first equation of model). The nonlinear relationship can be explored using the proposed approach, wherein the modification is implemented in the dependence of the response variable to the predictor variable (second equation of the model). Two next steps required to expand the model are to use two or more predictors, and to implement phylogenetic branch lengths as weight to update the priori distribution for each node in the phylogeny.

The model presented in this study is very flexible, as it may be adjusted to different relationship contexts, integrating ecology and evolution. The ability of a phylogenetic model to make more precise estimations of ecological responses for a given taxonomic group is an exciting subject in practical and theoretical ecology. Linking ecology and evolution to elucidate population and community structures must be a major focus, as these two study programs are complementary in both theory and subject. We recommend that the phylogeny‐based explicit logistic model presented may be further applied for different biological groups, given that this model is easily implemented and adds key evolutionary information for ecological studies.

## CONFLICT OF INTEREST

None declared.

## AUTHOR CONTRIBUTIONS

Godoy conceived the ideas and designed methodology; Godoy and Camargos collected the data; Godoy analyzed the data; Godoy, Camargos, and Lodi wrote the manuscript. All authors contributed critically to the drafts and gave final approval for publication.
