# Supplementary material for: When phylogeny and ecology meet: Modeling the occurrence of Trichoptera with environmental and phylogenetic data
Source: Ecol Evol. 2018 May 8;8(11):5313–22. doi: 10.1002/ece3.4031 (PMC6010749; doi:10.1002/ece3.4031)
Supplement: Supplementary file 1 [file ECE3-8-5313-s001.docx]

Supplementary Material 1

Final supertree for the Trichoptera individuals sampled in Cerrado streams and the number of the nodes in the phylogeny.


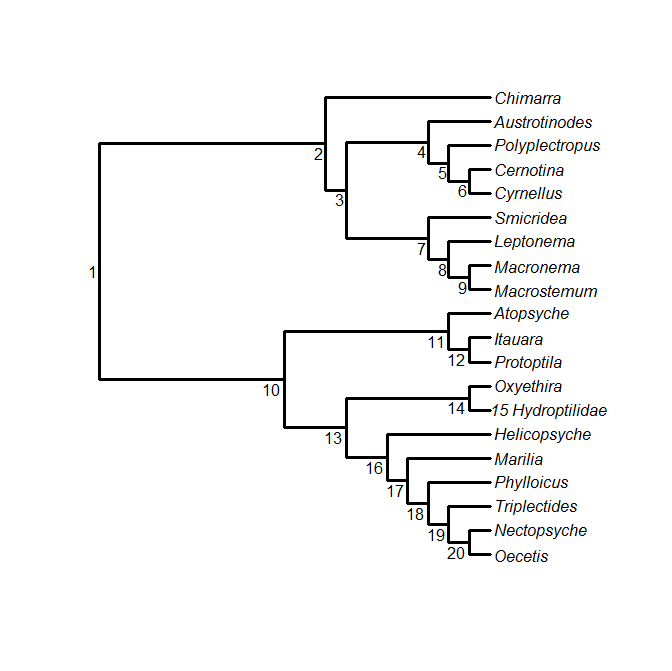


Supplementary Material 2

The phylogeny-based logistic regression model designed to estimate the species occurrence in the R software. The parameters are estimated considering the environmental information of species occurrence sites and the phylogenetic signal of close species.

#Data

library(BRugs)

occurrence<- data #The occurrence data is a binary matrix with the occurrence of the species, with sites in the rows and species in the columns.

x<-environmental variable #The environmental variable is a vector.

N<-number of nodes in the phylogeny #It is one numerical value.

S<-number of species

L<-number of sites

A<-parameter node id # A vector indicating which parameter each node in the phylogeny will use as a hyper-parameter.

B<-parameter specie id # A vector indicating which parameter each species will use as a hyper-parameter

richness<-rowSums(occurrence)

ldata<-list(occurrence=occurrence, x=x, N=N, S=S, L=L, A=A, B=B)

bugsData(ldata, fileName=file.path(tempdir(),"ldata.txt"))

#Parameters

parameters<-c('alpha', 'beta', 'beta.no', 'sigma')

iteration<-1000

burn<-1000

thin<-5

chain<-5

#Initials

initials<-function(){list(alpha=c(rep(sample(-50:50,1),S)), beta.no=c(rep(sample(-50:50,1),N)), tau.g=c(rep(sample(1:100,1),N)), beta=c(rep(sample(-50:50,1),S)))}

bugsInits(initials, numChains=1, fileName="initials.txt", digits=3, format="E")

#Linear logistic model

sink('model_phy.txt')

cat('

Model

{

x.med<-mean(x[])

beta.no[1] ~ dnorm(0.0,tau.g[1])

tau.g[1] ~ dgamma(0.001,0.001)

sigma[1] <- 1/sqrt(tau.g[1])

for(n in 2:N)

{

beta.no[n] ~ dnorm(beta.no[A[n]],tau.g[A[n]])

tau.g[n] ~ dgamma(0.001,0.001)

sigma[n] <- 1/sqrt(tau.g[n])

}

for(s in 1:S)

{

alpha[s] ~ dnorm(0.0,1.0E-6)

beta[s] ~ dnorm(beta.no[B[s]],tau.g[B[s]])

for(l in 1:L)

{

logit(p[l,s]) <- alpha[s] + beta[s]*(x[l]-x.med)

occurrence[l,s] ~ dbern(p[l,s])

}

}

}

',fill=TRUE)

sink()

fit.model<-BRugsFit(data="ldata.txt", inits=rep("initiais.txt", chain), para=parameters, modelFile='model_phy.txt', numChains=chain, nIter=iterations, nBurnin=burn, nThin=thin, coda=T)

#Extract the CODAS

size<-chain*(iterations/thin)

CODAS<-matrix(0,nrow=size, ncol=(2*S)+(2*N),

for(i in 1:((2*S)+(2*N)))

{

CODAS[,i]<-t(unlist(fit.model[,i]))

}
